# Tissue microarray analysis reveals a tight correlation between protein expression pattern and progression of esophageal squamous cell carcinoma

**DOI:** 10.1186/1471-2407-6-296

**Published:** 2006-12-22

**Authors:** Li-yan Xue, Nan Hu, Yong-mei Song, Shuang-mei Zou, Jian-zhong Shou, Lu-xia Qian, Li-qun Ren, Dong-mei Lin, Tong Tong, Zu-gen He, Qi-min Zhan, Philip R Taylor, Ning Lu

**Affiliations:** 1 Department of Pathology, Cancer Institute (Hospital), Peking Union Medical College, Chinese Academy of Medical Sciences, Beijing, China; 2 Genetic Epidemiology Branch, Division of Cancer Epidemiology & Genetics, National Cancer Institute, Bethesda, MD, USA; 3 State Key Laboratory of Molecular Oncology, Cancer Institute (Hospital), Peking Union Medical College, Chinese Academy of Medical Sciences, Beijing, China; 4 Department of Urology, Cancer Institute (Hospital), Peking Union Medical College, Chinese Academy of Medical Sciences, Beijing, China

## Abstract

**Background:**

The development of esophageal squamous cell carcinoma (ESCC) progresses a multistage process, collectively known as precursor lesions, also called dysplasia (DYS) and carcinoma *in situ *(CIS), subsequent invasive lesions and final metastasis. In this study, we are interested in investigating the expression of a variety of functional classes of proteins in ESCC and its precursor lesions and characterizing the correlation of these proteins with ESCC malignant progression.

**Methods:**

Fas, FADD, caspase 8, CDC25B, fascin, CK14, CK4, annexin I, laminin-5γ2 and SPARC were analyzed using immunohistochemistry on tissue microarray containing 205 ESCC and 173 adjacent precursor lesions as well as corresponding normal mucosa. To confirm the immunohistochemical results, three proteins, fascin, CK14 and laminin-5γ2, which were overexpressed in ESCC on tissue microarray, were detected in 12 ESCC cell lines by Western blot assay.

**Results:**

In ESCC and its precursor lesions, FADD, CDC25B, fascin, CK14, laminin-5γ2 and SPARC were overexpressed, while Fas, caspase 8, CK4 and annexin I were underexpressed. The abnormalities of these proteins could be classified into different groups in relation to the stages of ESCC development. They were "early" corresponding to mild and moderate DYS with overexpression of fascin, FADD and CDC25B and underexpression of Fas, caspase 8, CK4 and annexin I, "intermediate" to severe DYS and CIS with overexpression of FADD and CK14, and "late" to invasive lesions (ESCC) and to advanced pTNM stage ESCC lesions with overexpression of CK14, laminin-5γ2 and SPARC.

**Conclusion:**

Analyzing the protein expression patterns of Fas, FADD, caspase 8, CDC25B, fascin, CK14, CK4, annexin I, laminin-5γ2 and SPARC would be valuable to develop rational strategies for early detection of lesions at risk in advance as well as for prevention and treatment of ESCC.

## Background

Esophageal cancer is a common disease worldwide, especially in the north of China. Despite advances in therapy, the overall 5-year survival rate of esophageal cancer still remains less than 30% [1,2]. In contrast, 5-year survival rate of early esophageal cancer is higher than 90% [3]. Thus, prevention and early detection remain the best hope for a cure.

Squamous cell carcinoma is the most common histological type of esophageal cancer in China. A multistage process has been proposed for the evolution of esophageal squamous cell carcinoma (ESCC), in which normal squamous epithelia undergo a series of histological and genetic progression towards noninvasive precursor lesions, i.e., dysplasia (DYS) and carcinoma *in situ *(CIS), then towards invasive cancer, and, finally towards deep invasion and metastasis to lymph nodes and other organs. Understanding the genetic mechanisms underlying the progression is critical, because it would provide a clue for early detection and chemoprevention strategies.

We and others have recently explored the global expression profiles of ESCC with three different gene expression platforms (serial analysis of gene expression, oligonucleotide microarray, and cDNA microarray) [4-10], and have identified a multitude of genes that were overexpressed or underexpressed on transcriptional level in ESCC, compared with normal epithelia/mucosa, including Fas, FADD, caspase 8, CDC25B, fascin, CK14, CK4, annexin I, laminin-5γ2 and SPARC, encompassing a variety of functional classes. The results in these studies on the dysregulation of most of the above genes such as CK14, CK4 and SPARC were concordant [4-10].

Although cDNA microarray has been successfully used to explore the gene expression in ESCC, it is difficult to be used in the precursor lesions due to the difficulty to obtain the dysplastic cells accurately. Only one study examined the gene expression in precursor lesions and different pTNM stages of ESCC using cDNA microarray, and showed complex alterations of gene expression in each stage of tumorigenesis [8]. At present, however, little has been known about the alterations on protein level in different stages.

The method of tissue microarray (TMA), which could confirm the results of cDNA microarray on protein level, has been popularly used for gene expression profiling in many types of cancer including ESCC, but rarely in the consecutive stages of carcinogenesis, especially in the precursor lesions. TMA is more accurate and convenient than cDNA microarray for precursor lesions of ESCC. We have constructed TMA comprised of tissues of a variety of pTNM stages of ESCC, a variety of grades of precursor lesions and normal epithelia/mucosa. In this study, we examined the expression of the 10 protein markers mentioned above in ESCC and the precursor lesions compared with normal epithelia/mucosa using TMA-based immunohistochemistry. Based on the results of TMA-based immunohistochemistry, we detected the expression of three overexpressed proteins, fascin, CK14 and laminin-5γ2 in 12 ESCC cell lines by Western blot assay to confirm the immunohistochemical results.

## Methods

### Sample collection and TMA construction

205 esophageal cancer specimens used in this study were originated from patients who had received esophagectomy without radiotherapy and chemotherapy before operations in Cancer Institute (Hospital), Peking Union Medical College, Chinese Academy of Medical Sciences from June, 2001 to June, 2002. Among these patients, 163 were male and 42 were female. The age of them ranged from 33 to 83 years, with the mean of 58.3. 181 cancer tissues combined with adjacent mucosa and distant normal mucosa were cut immediately after surgery, fixed in 80% ethanol and embedded in paraffin. The other 24 samples were obtained from the archival paraffin blocks of the Department of Pathology, which had been fixed in 10% formalin. All the specimens were cut into 4 μm sections and stained by conventional HE-staining method. All the cases were diagnosed as squamous cell carcinoma by two authors (SZ and LR). Among them, 48 were well differentiated, 99 moderately differentiated and 58 poorly differentiated. The cases were staged according to the UICC 1997 pTNM criteria. The 24 cases from the archives were early stage lesions (I), selected consecutively, and the other 181 cases were advanced stage ones (IIA 64, IIB 18, III 90 and IV 9), selected randomly. 173 precursor lesions in the adjacent mucosa were selected and classified as 22 mild DYS, 25 moderate DYS, 51 severe DYS, and 75 CIS.

The follow-up data were mainly gathered from clinical notes or via phone call and mail. Disease-free survival time was recorded by the number of months from the date of surgery to the date when any of the following events happened: recurrence, metastasis, or death of ESCC. We had excluded the patients who were in stage I, who suffered from severe post-operative complications, who died of other tumors or other causes, and who had not been definitely followed up. The patients were followed up for a maximum period of 60 months and mean of 27 months. This study was approved by the review board of Cancer Institute (Hospital), Peking Union Medical College, Chinese Academy of Medical Sciences, and informed consent was obtained from all patients.

For TMA construction, representative areas containing morphologically representative ESCC, normal epithelia/mucosa and defined precursor lesions were circled on the glass slides and used as a template. The TMA was constructed using a manual Tissue Arrayer (Beecher Instruments, Silver Spring, MD) as previously described [11]. For each lesion, more than two 0.6-mm cores were punched from the circled regions in the donor block randomly (but not from specific areas) and arrayed on the recipient block to ensure the representation and to avoid missing information due to loss of tissue cores.

### Immunohistochemistry and assessment

The standard streptavidin peroxidase (SP) method was employed for immunostaining. Briefly, sections were cut at 4 μm from the TMA blocks. After dewaxed in xylene and rehydrated in alcohol and distilled water, antigen retrieval was performed by micro-wave oven heating (10 minutes) at middle power in 0.01 M sodium citrate buffer (pH 6.0) or incubated with protease XXIV (Biogenex, San Ramon, CA) (10 minutes) at room temperature (Table 1). Then, sections were incubated with 3% hydrogen peroxide for 10 minutes to block endogenous peroxidase activity. Nonspecific staining was blocked by 10% normal goat serum (Vector Laboratories Inc, Burlingame, CA) for 10 minutes. The primary antibodies used, as well as their respective dilutions, incubation conditions and sources, were shown in Table 1. The bound antibody was detected then with biotinylated anti-mouse/rabbit IgG(H+L) (Vector Laboratories Inc, Burlingame, CA) for 10 minutes and horseradish peroxidase streptavidin (Beijing Zhongshan Biotech, Beijing, China) for 10 minutes. 3,3'-diaminobenzidine (Maixin Biotech, Fuzhou, Fujian, China) was used as the chromogen. Slides were lightly counterstained with hematoxylin. In control experiments, the primary antibody was replaced by PBS. Internal positive controls were available on the TMA itself, for example, basal cells in the normal squamous cell epithelia expressed fascin and CK14; normal sqaumous cell epithelia expressed Fas and CK4; superficial layers of normal sqaumous cell epithelia expressed caspase 8. For all immunohistochemical stains, the pattern of staining in normal epithelia/mucosa was considered to be the baseline for comparison with precursor lesions and ESCC.

Because the staining intensity of Fas, FADD, caspase 8, CDC25B, fascin, CK14, CK4 and annexin I was not uniform among different lesions, we scored these proteins with the criteria combined intensity with the rate of positive cells. First, the intensity was graded as follows: 0, negative; 1, weak; 2, moderate; 3, strong. Second, the rate of positive cells was graded: 0, <5%; 1, 5~25%; 2, 26~50%; 3, 51%~75%; 4, >75%. A final score was achieved by multiplication of the two scores above. Scores of 0~4 were defined as "negative expression" (-); scores of 5~8 as "weakly positive expression" (+), and scores of 9~12 as "strongly positive expression" (++) [12].

For laminin-5γ2, the expression levels were graded on a scale as follows: negative (-), no positive cells; weakly positive (+), cluster(s) of positive cells present, but accounting for less than 30%; strongly positive (++), cluster(s) of positive cells more than 30% [13].

The staining of SPARC was graded in the stroma and cancer cells/normal epithelia separately. The extent of staining in tumor stroma/normal mucosa stroma was recorded according to the criteria of Koukourakis MI *et al*. as follows: negative (-), no positive fibroblasts; weakly positive (+), less than 50% of fibroblasts positive; strongly positive (++), more than 50% of fibroblasts positive [14]. However, Koukourakis MI *et al*. only scored the stroma by semi-quantitative method, but not the tumor cells/normal epithelia. We modified the criteria and also scored the tumor cells/normal epithelia semi-quantitatively as follows: negative (-), no positive cells; weakly positive (+), less than 30% cells positive; strongly positive (++), more than 30% cells positive. We selected 30% as cut-off, like the scoring criteria of laminin-5γ2, but not 50%, due to the low expression level of SPARC in tumor cells.

The immunohistochemically stained sections were reviewed independently by two pathologists (SZ and LR). As to the discordant cases, unanimous concordance was reached after revising and discussing.

### Cell culture

Human ESCC cell lines, EC9706, TE12, COLO-680N, KYSE510, KYSE450, KYSE410, KYSE180, KYSE150, KYSE140, KYSE70, KYSE30 and YES2 were cultured in RPMI 1640 medium supplemented with 10% fetal bovine serum at 37°C under 5% CO_2 _and saturated moisture.

### Western blot

Cultured cells were collected and dissolved in lysis buffer containing 50 mM Tris-Cl (pH 8.0), 150 mM NaCl, 0.1% SDS, 100 μg/ml PMSF, 2 μg/ml aprotinin, 2 μg/ml leupeptin, 1% NP-40 on ice for 30 minutes. After centrifugation, the supernatant was collected for Western blot analysis. Briefly, 50 μg cellular proteins were loaded onto 12% SDS-PAGE gels. After electrophoresis, the proteins were transferred to nitrocellulose membranes. The membranes were blocked in 5% nonfat milk, and then incubated with primary antibody, fascin (1:5000, diluted with 5% nonfat milk), CK14 (1:500, diluted with 5% nonfat milk) or laminin-5γ2 (1:500, diluted with PBS) for 3 hours at room temperature. Sources of antibodies were shown in Table 1. The membranes were washed with PBST (PBS with 0.1% tween-20) and then incubated with horseradish peroxidase-conjugated anti-mouse antibody at 1:4000 in 5% nonfat milk. The signals were developed by an ECL kit (Amersham, Arlington Height, IL) [15,16].

### Trans-well cell migration assay

Cell migration was examined with a chemotaxis chamber (Neuro Probe Inc. Gaithersburg, MD). Cells (1 × 10^4^) in 50 μl culture medium were added to the upper chamber of the device, and the lower chamber was filled with 30 μl medium containing 5 μg/ml fibronectin (Sigma, St. Louis, MO). A polycarbonate membrane with a pore size of 8 μm was placed between the two chambers. The cells were allowed to migrate at 37°C in a 5% CO_2 _humidified incubator for 6 hours. Non-migrated cells on the upper surface were carefully removed with a cotton swab. The filters were then fixed in methanol for 10 minutes and stained with Giemsa solution. Migration was quantified by counting the migrated cells in 10 random high-powered fields per filter. The experiments were repeated three times [15].

### Statistical analysis

χ^2 ^test performed with SPSS 10.0 for Windows (SPSS, Chicago, IL, USA) was used to compare the expression of the proteins among the consecutive stages of carcinogenesis, including normal epithelia/mucosa, different grades of precursor lesions and different pTNM stages of ESCC and to assess the correlations between the expression of the proteins in ESCC and clinicopathological characteristics, including sex, age and differentiated grades. Survival analysis was carried out using Kaplan-Meier method with log-rank test. *P *value of less than 0.05 was considered statistically significant.

## Results

### Protein expression in ESCC and the precursor lesions detected by immunohistochemistry

A summary for expression of the proteins in various histological categories of esophageal lesions was shown in Table 2. In ESCC and the precursor lesions, fascin, FADD, CDC25B, CK14, laminin-5γ2 and SPARC were overexpressed, while Fas, caspase 8, CK4 and annexin I were underexpressed, compared with normal epithelia/mucosa, which were consistent with previous studies on mRNA level [4-10].

**Figure 1:**
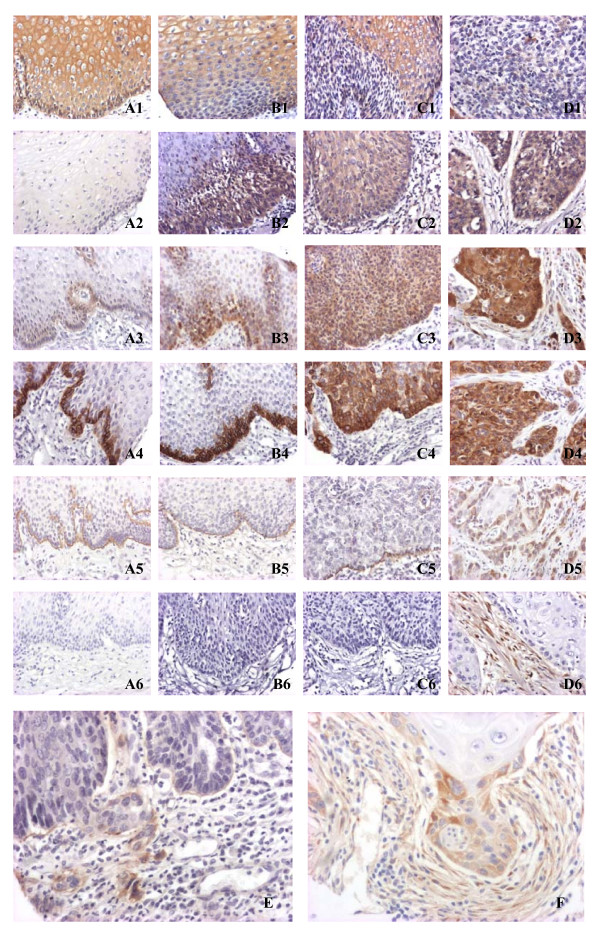
**Representative photographs of immunohistochemical features of the proteins in different lesions**. The proteins of interest are designated in rows, and the esophageal histological categories, normal esophageal mucosa (**A**) and various histological categories of lesions, i.e., mild DYS (**B**), severe DYS (**C**) and ESCC (**D**), are designated in columns. **A1-D1**, strongly positive expression of Fas in normal epithelia, but negative in mild, severe DYS and ESCC. **A2-D2**, negative expression of FADD in normal epithelia, but strongly positive in mild, severe DYS and ESCC. **A3-D3**, positive expression of fascin only in basal cells of normal epithelia, and strongly positive in mild, severe DYS and ESCC.**A4-D4**, positive expression of CK14 only in basal cells of normal epithelia, negative in mild DYS, but strongly positive in severe DYS and ESCC. **A5-D5**, negative expression of laminin-5γ2 in cytoplasm of normal epithelia and mild, severe DYS with positive expression only in basement membrane, but strongly positive in cytoplasm of ESCC, especially at the tumor-stroma interface. Moreover, positive expression of laminin-5γ2 can be seen in the budding tumor cells of the microinvasive cancer (**E**). **A6-D6**, negative expression of SPARC in the epithelia and stroma in normal mucosa and mild, severe DYS, but prominent SPARC expression by stromal fibroblasts in a background of negative cancer cell reactivity in ESCC. Moreover, SPARC expression can be seen in the cytoplasm of the juxta-stromal tumor cells in a few cases (**F**). (original magnification, ×200)

The representative immunohistochemical features of the proteins were shown in Figure 1. The localization of positivity of the following proteins was interesting. Caspase 8 was strongly positive in superficial layers of normal epithelia, but negative in all basal, superbasal cells of normal epithelia, all precursor lesions and all ESCC. Fascin and CK14 were positive only in basal cells of normal epithelia, but diffusely positive in ESCC. Laminin-5γ2 was negative in cytoplasm of normal epithelia and precursor lesions, with basement membrane positive only, but it was strongly positive in cytoplasm of cancer cells, especially the cancer cells at the tumor-stroma interface in ESCC. Moreover, positive expression of laminin-5γ2 could be seen in the budding tumor cells of the microinvasive cancer. SPARC was negative in most normal mucosa and precursor lesions, but strongly positive in stromal fibroblasts in most ESCC. High levels of cytoplasmic SPARC in cancer cells could be seen in a few cases, and the immunoactivity was detected mainly in the cancer cells localized at the tumor-stroma interface, similar to the pattern of laminin-5γ2. So it could be seen in most ESCC that SPARC was prominently expressed by stromal fibroblasts in a background of negative cancer cell reactivity.

**Figure 2:**
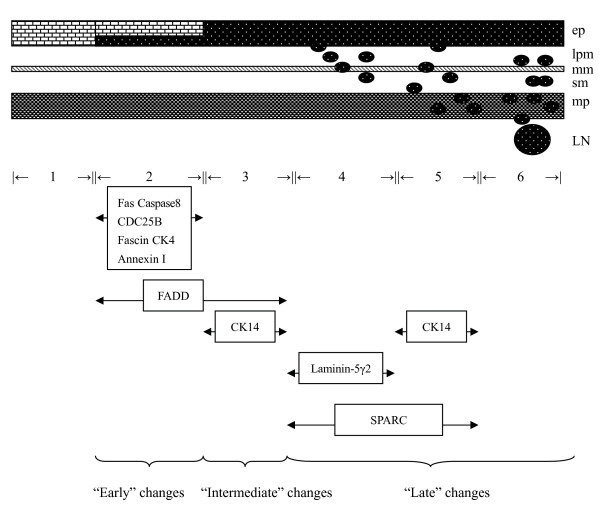
**An outline of our current understanding of protein expression changes in multistep progression of ESCC**. The "early" changes are those occurring at the transition from normal epithelia to mild and moderate DYS, the "intermediate" changes are those occurring at transition from mild and moderate DYS to severe DYS and CIS, and the "late" changes are those occurring at the transition from severe DYS and CIS to invasive lesions or at the transition from early stage ESCC to late stage. (1, normal epithelia/mucosa; 2 mild and moderate DYS; 3, severe DYS and CIS; 4, ESCC stage I; 5, ESCC stage IIA and IIB; 6, ESCC stage III and IV; ep, epithelium; lpm: lamina propria mucosa; mm: muscularis mucosa; sm: submucosa; mp, muscularis propria; LN, lymph node.)

Most of the abnormalities of these proteins occurred at mild and moderate DYS (overexpression of fascin, FADD, CDC25B and underexpression of CK4, annexin I, Fas, caspase 8), which were defined as "early" changes; some occurred at severe DYS and CIS (overexpression of CK14 and FADD), defined as "intermediate" changes; some occurred at the transition from severe DYS and CIS to invasive lesions or at the transition from early stage ESCC to late stage (overexpression of CK14, laminin-5γ2 and SPARC), defined as "late" changes. Figure 2 demonstrated our current model of abnormalities in the progression of ESCC.

In addition, we had detected the expression patterns of mutant p53 protein in the TMA (see additional file 1).

### Association between protein expression and clinicopathologic characteristics

The expression of SPARC in stromal fibroblasts was correlated with sex, the expression of CK4 was correlated with age and the expression of Fas, FADD, fascin, CK14, laminin-5γ2 and SPARC in stromal fibroblasts was correlated with the differentiated grades (Table 3).

### Survival analysis

**Figure 3:**
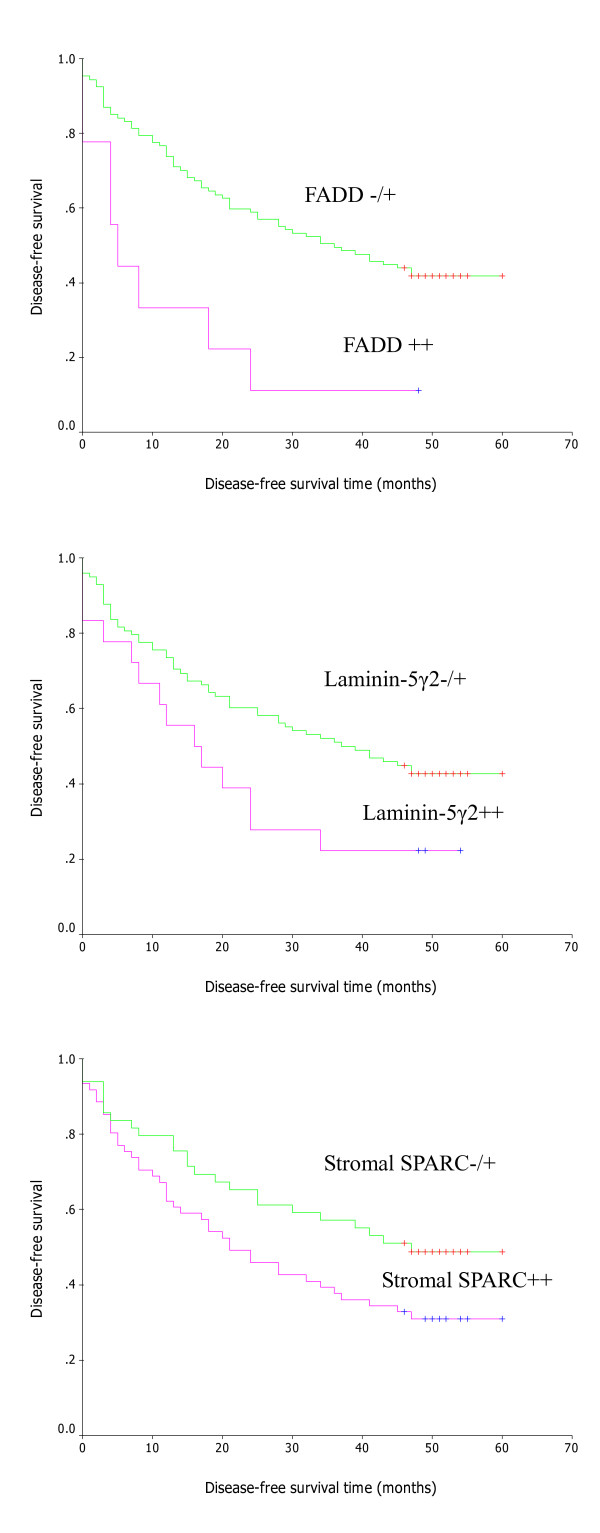
**Representative survival curves of ESCC**. Kaplan-Meier survival curves of patients with negative/weak or strong expression of FADD, laminin-5γ2 and SPARC.

Survival analysis using the log-rank test showed that the expression of FADD, laminin-5γ2 and SPARC was significantly correlated with disease-free survival (*P *= 0.0022, *P *= 0.0380, and *P *= 0.0494, respectively) (Table 4 and Figure 3).

### The expression of fascin, CK14 and laminin-5γ2 in ESCC cell lines

**Figure 4:**
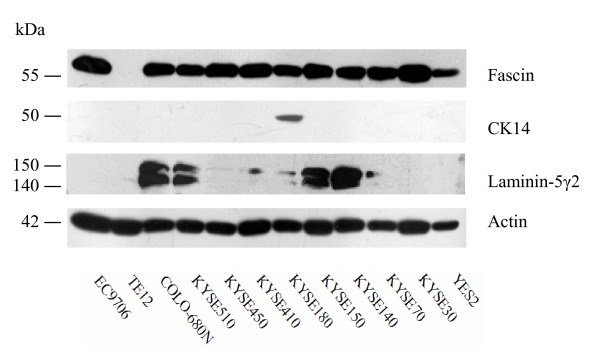
**Western blot of fascin, CK14 and laminin-5γ2 in 12 ESCC cell lines**. High expression of fascin was detected in almost all cell lines, but not in cell line TE12. Expression of CK14 was detected in cell line KYSE180. High expression of laminin-5γ2 was detected in cell lines, COLO-680N, KYSE510, KYSE150 and KYSE140, and moderate expression was detected in KYSE410, KYSE180 and KYSE70.

High expression of fascin was detected in almost all ESCC cell lines utilized in our study, but not in cell line TE12. Expression of CK14 was seen only in KYSE180. High expression of laminin-5γ2 was detected in COLO-680N, KYSE510, KYSE150, KYSE140, moderate in KYSE410, KYSE180, KYSE70 and negative in others (Figure 4). We analyzed the correlation of the expression of these three proteins with the results of trans-well cell migration assay, because cell-migration ability was closely associated with the potential of invasion and metastasis [15]. According to the number of migrated cells in trans-well cell migration assay, the cell lines were classified into two groups. Six cell lines, EC9706, KYSE450, KYSE180, COLO-680N, KYSE410, KYSE510, which were classified as "strong" migration group, had more migrated cells than the other six cell lines, YES2, KYSE150, KYSE140, KYSE30, KYSE70, TE12, classified as "weak" migration group. Among them, EC9706 had the most migrated cells and TE12 had the least migrated cells, which were defined as the "strongest" and the "weakest", respectively. The correlation of the expression of these three proteins with the results of trans-well cell migration assay [15] and the correlation of the expression of these three proteins with the differentiation of the primary tumor of these cell lines [16,17] were shown in Table 5. The cell line with negative expression of fascin displayed the weakest migration in trans-well test, and the cell lines with high and moderate expression of laminin-5γ2 displayed stronger migration phenotypes than those with negative expression of laminin-5γ2.

## Discussion

Tumorigenesis is a multistep process that may involve many genes. Analyzing alterations of gene expression profiles in different stages of neoplasia is a necessary step towards establishing the diagnostic, prognostic, therapeutic and preventive potential of each related gene. According to gene expression data previously established, we further examined the immunohistochemical labeling of 10 protein markers using the TMA composed of different pTNM stages of ESCC, different grades of precursor lesions and normal mucosa.

### Apoptosis related proteins (Fas, FADD, caspase 8)

Fas, a member of the tumor necrosis factor receptor superfamily, is a transmembrane protein which can bind its ligand (FasL) and induce apoptosis by triggering a cascade of downstream effector caspases via FADD and caspase 8 [18]. Functional impairment of the apoptotic pathway is associated with the development and progression of malignancies by evading immune surveillance.

Downregulation of Fas has been demonstrated previously in ESCC and its precursor lesions [19]. We further demonstrated loss of Fas expression occurred at the phase as early as mild and moderate DYS.

To our knowledge, detailed analysis of FADD protein expression has not been performed in ESCC and its precursor lesions. In the present study, we demonstrated that FADD was overexpressed in ESCC compared with normal esophageal epithelia, consistent with the result on mRNA level in cDNA microarray study [4]. Moreover, FADD was overexpressed at the transition from normal epithelia to mild and moderate DYS and at the transition from mild and moderate DYS to severe DYS & CIS as an "early" and "intermediate" event.

At present, there was little information about the expression of caspase 8 in ESCC and its precursor lesions. In this study, we demonstrated that caspase 8 was strongly positive in the mature superficial layers of normal esophageal epithelia, but negative in the basal and suprabasal layers of normal esophageal epithelia, all precursor lesions and all invasive cancers.

### Cell cycle regulatory proteins (CDC25B)

Alterations in cell-cycle regulatory genes are commonly found in human cancers. CDC25B is a dual specificity phosphatase and can positively control the G2-M transition [20].

Overexpression of CDC25B protein in ESCC has been reported previously [9,21]. In the present study, we further investigated the expression of CDC25B in different grades of DYS, CIS as well as in different pTNM stages of ESCC. We demonstrated that CDC25B was overexpressed in ESCC and the precursor lesions, and CDC25B overexpression was an "early" event, occurring at mild and moderate DYS.

### Cytoskeleton and related proteins (fascin, CK14 and CK4)

The cytoskeleton is a complex network of protein filaments that extends throughout the cytoplasm of the eukaryotic cells. It includes 3 types of protein filaments: actin filaments, microtubules, and intermediate filaments. Changes in cytoskeleton components or associated binding proteins may be implicated in the progression and metastasis of tumors.

Fascin is a highly conserved actin-bundling protein. The overexpression of fascin induces membrane protrusions and cell motility. Immunoreactivity for fascin has been reported in various malignancies [22,23]. Moreover, upregulation of fascin has been considered as an "intermediate" event in pancreatic carcinoma progression, uncommon in early intraepithelial neoplasia lesions, but substantially upregulated in advanced intraepithelial neoplasia lesions and nearly universal in invasive cancers [24]. In the current study, we found that fascin overexpression occurred at mild and moderate DYS as an "early" event, and was nearly universal in precursor lesions and invasive cancers.

Yashimoto et al. demonstrated that all of the 33 cell lines tested expressed fascin protein, and a fascin-overexpressed cell line, KYSE170, decreased its motile and invasive properties after down-regulation of fascin expression by using vector-based small interfering RNA [25]. In the present study, high expression of fascin was detected in almost all cell lines, but not in cell line TE12, which grew the slowest and displayed the weakest migration [15]. These suggested that fascin might play an important role in the progression of ESCC.

Cytokeratins are a family of intermediate filament proteins typically found in epithelial cells. Based on the isoelectric point and molecular weight, human cytokeratins have been classified numerically as cytokeratins 1–20. Carcinoma cells retain the ability to produce the cytokeratins of their normal progenitor cells and may also gain the ability to develop new expression types.

Previous studies have demonstrated that CK14 protein was expressed in squamous cell carcinoma regardless of origin and degree of differentiation, and could be used as a useful marker in diagnosis of squamous cell carcinoma [26]. Takahashi H *et al*. demonstrated that CK14 selectively labeled the basal cells of normal esophageal epithelia and also labeled all DYS, all CIS and all ESCC. [27]. We further found that dysregulation of CK14 might be an "intermediate" and "late" event in ESCC progression, uncommon in mild and moderate DYS, but substantially upregulated in severe DYS and CIS and more universal in relatively late stages of invasive cancers.

Expression of CK14 was only seen in cell line KYSE180. In contrast, CK14 was expressed in most ESCC tissues. Similar phenomenon could be seen in oral cancer. CK14 was overexpressed in most oral squamous cell carcinoma tissue [28], but was lost or downregulated in oral squamous cell carcinoma cell lines [29,30]. Moreover, it had been demonstrated that the cytokeratin expression pattern changed in normal esophageal epithelial cells during culture [31]. These suggested that the expression of CK14 in ESCC cell lines might decrease during culture and passage.

CK4 is expressed during the development of stratified epithelia from one-layered polar epithelia and continues to be expressed in several adult nonepidermal stratified epithelia including esophageal epithelia. Low levels of CK4 expression have been detected in squamous cell carcinoma of the tongue, larynx and sinonasal tract [32,33]. In nonkeratinizing squamous cell carcinoma of sinonasal tract associated with a Schneiderian papilloma, CK4 was expressed in the papilloma but not in the carcinoma from the same cases [32]. So downregulation of CK4 might occur during malignant transformation of these tumors. In the current study, CK4 was positively expressed in normal esophageal epithelia, but obviously decreased in ESCC [33]. We found CK4 downregulation occurred at mild and moderate DYS as an "early" event, which was first reported.

### Cell signaling protein (annexin I)

Annexin I is a member of the annexin family, which can bind (annex) to cellular membranes in a calcium-dependent manner. Recent data have shown that annexin I might be an cell signaling protein and be implicated in the signaling processes of inflammation, differentiation, apoptosis, coagulation, immune response and proliferation [34].

The role of annexin I in tumor biology is attracting growing interests. This is partly because characteristic distribution and expression of annexin I have been found in different normal tissues, and dysregulation of annexin I expression has been described in a variety of cancerous and precancerous lesions [35-38]. Paweletz *et al*. demonstrated that annexin I was downregulated in all ESCC, compared with patient-matched normal epithelia, and suggested that annexin I might be an essential component for maintenance of the normal esophageal epithelial phenotype and its loss might be correlated with tumorigenesis. In addition, they analyzed 11 patient-matched cases with different grades of lesions with the method of LCM (laser capture microdissection) and Western blot, and demonstrated that downregulation of annexin I was an "early" event in the progression of ESCC, occurring either at the junction between high-grade DYS and invasive phenotypes or at the low-grade to high-grade DYS transition [37]. In the current study, dysregulation of annexin I expression occurred at the transition from normal epithelia to mild & moderate DYS, but not at the transition from severe DYS & CIS to ESCC as a very "early" event. The discordance was perhaps due to the different methods and the different cases utilized in the studies. Anyway, the dysregulation of annexin I occurred as a relatively "early" change.

### Extracellular matrix protein (laminin-5γ2) and matricellular protein (SPARC)

Laminins are a group of extracellular matrix proteins localized at the basement membrane where they are involved in cell adhesion, migration, proliferation, and differentiation. Laminin-5 is a heterotrimeric glycosylated protein that belongs to the laminin family and is formed by α3β3γ2 chains, and the γ2 chain is unique to laminin-5 [39]. Overexpression of the γ2 chain has been reported in several malignancies, such as gastric, tongue, colon, cervical cancer and melanoma. In addition, it has been demonstrated to be implicated in cancer invasion and metastasis [40-42].

In normal esophageal mucosa, we found that immunoreactivity for laminin-5γ2 was strong in basement membrane, but negative in cytoplasm of epithelial cells. We also found that immunoreactivity in basement membrane was strong in specimens fixed in ethanol, but weak or negative in those fixed in formalin, which was similar to the results of Aoki S *et al*. [43]. In our present study, only 11.9% of mild & moderate DYS and 25.4% of severe DYS & CIS were positive (mainly weakly positive), with a tendency of correlation between the grade of precursor lesions and laminin-5γ2 immunoreactivity in cytoplasm, consistent with previous studies in other tissues [42,44,45]. As far as we know, this is the first report investigating the laminin-5γ2 chain staining pattern in esophageal preinvasive lesions. In ESCC, strong expression was seen in cytoplasm of cancer cells in most cases, especially at the tumor-stroma interface. So the dysregulation of laminin-5γ2 chain predominantly occurred at the transition of preinvasive lesions to ESCC as a "late" event.

A few previous studies have investigated the possibility of using laminin-5γ2 chain expression as a preinvasive and microinvasive marker [42]. In our study, positive signals of laminin-5γ2 chain were seen in all the 3 microinvasive lesions found occasionally (Fig 1E) and in all the 4 lesions suspicious for invasion. The findings suggested that laminin-5γ2 chain could be a microinvasive marker and would facilitate the identification of invasive lesions that are difficult or impossible to identify on routinely stained histological sections.

The cell lines with high and moderate expression of laminin-5γ2 displayed stronger migration phenotypes in trans-well test than those with negative expression of laminin-5γ2 [15]. These suggested that laminin-5γ2 might play an important role in the progression of ESCC.

Matricellular proteins comprise a nonhomologous group of extracellular regulatory macromolecules that mediate cell-matrix interactions, but may not contribute significantly to extracellular matrix structure. Therefore, the matricellular proteins are different from the traditional extracellular matrix proteins including laminin, all of which are adhesive proteins and contribute to the structural stability of the extracellular matrix.

SPARC (secreted protein, acidic and rich in cysteine) is a prototype of the nonstructural matricellular proteins. Increasing evidence in the literature have suggested that overexpression of SPARC might play a role in many types of tumor, and both tumor and stromal cells within tumor have been shown to express SPARC [46,47]. In the current study, cytoplasmic SPARC in cancer cells was seen in a few cases, and in these cases, immunoactivity was detected in cancer cells mainly localized at the tumor-stroma interface, similar to the pattern of laminin-5γ2. In contrast to cancer cells, stromal fibroblasts were frequently and strongly reactive for SPARC. So it could be seen in most ESCC that SPARC was prominently expressed by stromal fibroblasts in a background of negative cancer cell reactivity. Moreover, the immunoactivity of stromal fibroblasts was higher in late stage ESCC than relatively early stage ones. However, SPARC was negative in most normal mucosa and precursor lesions. Thus, its dysregulation might be a "late" step of carcinogenesis.

## Conclusion

In summary, we have examined 10 proteins encompassing a variety of functional classes in ESCC and its precursor lesions using a TMA-based approach. We distinguished the different protein expression patterns among "early", "intermediate" and "late" progression, which would hopefully lead to rational early-detection strategies and prognosis strategies in patients at risk of developing esophageal cancer, as well as of advancing. Thereby, the proteins that displayed specific changes in ESCC and its precursor lesions could be used as markers to guide rational "individual" treatment.

## Competing interests

The author(s) declare that they have no competing interests.

## Authors' contributions

LX, constructed tissue microarray, carried out immunohistochemistry and Western blot, and drafted the manuscript

NH, participated in the design and data analysis

YS, participated in cell culture and Western blot

SZ, read the H&;E slides and scored the immunostained slides

JS, participated in immunohistochemistry

LQ, participated in immunohistochemistry

LR, read the H&E slides and scored the immunostained slides

DL, helped to collect the specimens and the follow-up data

TT, participated in Western blot, and helped to interpret the results

ZH, helped to revise the manuscript

QZ, helped to interpret the results of Western blot and revise the manuscript

PT, participated in the design

NL, conceived the study, participated in its design and helped to revise the manuscript

All authors read and approved the final manuscript.

## Pre-publication history

The pre-publication history for this paper can be accessed here:



## Supplementary Material

Additional file 1 **The expression of mutant p53 protein in the tissue microarray**. The expression of mutant p53 protein was an "early" event, occurring at mild and moderate DYS. Click here for file

## Figures and Tables

**Table 1:** Antibodies

Antibody	Clone	Dilution &condition	Pretreatment	Source
Fas (C-20)		1:200 RT1 h	MWO-CB	Santa Cruz Bio, Santa Cruz, CA, USA
FADD	64A6	1:20 4°C overnight	MWO-CB	Novacastra, Newcastle upon Tyne, UK
Caspase 8	8CSP01	1:200 4°C overnight	MWO-CB	NeoMarkers, Fremont, CA, USA
CDC25B		1:50 RT1 h	MWO-CB	Cell Signaling, Beverly, MA, USA
Fascin	55K-2	1:50 RT1 h	MWO-CB	Dako Corp, Carpinteria, CA, USA
CK14	LL002	1:50 RT1 h	MWO-CB	NeoMarkers, Fremont, CA, USA
CK4	6B10	1:50 4°C overnight	MWO-CB	Novacastra, Newcastle upon Tyne, UK
Annexin 1	29	1:100 RT1 h	MWO-CB	BD Transduction Laboratories, Lexington, KY
Laminin-5γ2	D4B5	1:50 RT1 h	Protease XXIV	Chemicon, Temecula, CA, USA
SPARC	15G12	1:50 RT1 h	MWO-CB	Novacastra, Newcastle upon Tyne, UK

RT, room temperature

MWO-CB, microwave oven heating in citrate buffer.

**Table 2:** Summary of immunohistochemical abnormalities in different lesions

			Precursor lesions*		ESCC*			*P-value*					
			
Protein		Normal	Low#	High##	I	IIA/IIB	III/IV	*P*^a^	*P*^b^	*P*^c^	*P*^d^	*P*^e^	*P*^f^
	-	22(11.8%)	28(65.1%)	71(62.8%)	14(77.8%)	42(55.3%)	56(57.7%)						
Fas	+	89(46.6%)	13(30.2%)	31(27.4%)	4(22.2%)	30(39.5%)	31(32.0%)	<0.001	<0.001	0.583	0.295	0.184	0.355
	++	76(40.6%)	2(4.7%)	11(9.7%)	0	4(5.3%)	10(10.3%)						

	-	155(84.7%)	15(38.5%)	28(23.5%)	1(5.6%)	36(44.4%)	58(59.2%)						
FADD	+	25(13.7%)	20(51.3%)	56(47.1%)	11(61.1%)	40(49.4%)	28(28.6%)	<0.001	<0.001	0.032	0.212	<0.001	0.013
	++	3(1.6%)	4(10.3%)	35(29.4%)	6(33.3%)	5(6.2%)	12(12.2%)						

	-	72(39.1%)	40(100%)	114(100%)	18(100%)	81(100%)	98(100%)						
Caspase 8	+	76(41.3%)	0	0	0	0	0	------	------	------	------	------	------
	++	36(19.6%)	0	0	0	0	0						

	-	102(53.4%)	9(21.4%)	47(38.5%)	3(16.7%)	30(37.0%)	30(30.6%)						
CDC25B	+	76(39.8%)	26(61.9%)	45(36.9%)	5(27.8%)	43(53.1%)	59(60.2%)	<0.001	0.001	0.018	0.021	<0.001	0.618
	++	13(6.8%)	7(16.7%)	30(24.6%)	10(55.6%)	8(9.9%)	9(9.2%)						

	-	152 ** (82.2%)	9(20.5%)	14(11.7%)	2(11.8%)	16(19.8%)	16(16.3%)						
Fascin	+	17(9.2%)	23(52.3%)	62(51.7%)	6(35.3%)	29(35.8%)	43(43.9%)	<0.001	<0.001	0.272	0.402	0.701	0.203
	++	16(8.6%)	12(27.3%)	44(36.7%)	9(52.9%)	36(44.4%)	39(39.8%)						

	-	159**(86.9%)	30(78.9%)	65(59.0%)	13(68.4%)	29(35.8%)	33(34.0%)						
CK14	+	16(8.7%)	5(13.2%)	29(26.4%)	3(15.8%)	12(14.8%)	24(24.7%)	<0.001	0.436	0.035	0.612	0.019	0.241
	++	8(4.4%)	3(7.9%)	16(14.5%)	3(15.8%)	40(49.4%)	40(41.2%)						

	-	5(2.8%)	39(86.7%)	85(70.2%)	18(94.7%)	39(48.8%)	49(49.5%)						
CK4	+	123(68.0%)	6(13.3%)	34(28.1%)	1(5.3%)	39(48.8%)	46(46.5%)	<0.001	<0.001	0.020	0.079	0.001	0.832
	++	53(29.3%)	0	2(1.7%)	0	2(2.5%)	4(4.0%)						

	-	1(0.5%)	24(53.3%)	52(43.3%)	10(52.6%)	44(55.7%)	58(58.6%)						
Annexin 1	+	48(26.0%)	16(35.6%)	46(38.3%)	6(31.6%)	30(38.0%)	30(30.3%)	<0.001	<0.001	0.402	0.749	0.391	0.377
	++	136(73.5%)	5(11.1%)	22(18.3%)	3(15.8%)	5(6.3%)	11(11.1%)						

	-	181***(100%)	37(88.1%)	88(74.6%)	6(37.5%)	26(32.1%)	22(22.4%)						
Laminin-5γ2	+	0	5(11.9%)	29(24.6%)	6(37.5%)	46(56.8%)	56(57.1%)	------	------	0.181	<0.001	0.227	0.142
	++	0	0	1(0.8%)	4(25%)	9(11.1%)	20(20.4%)						

	-	167(96.0%)	40(95.2%)	116(97.5%)	10(71.4%)	13(18.3%)	11(11.6%)	<0.001	0.651	0.606	<0.001	<0.001	0.125
SPARC in stromal fibroblasts	+	5(2.9%)	2(4.8%)	3(2.5%)	3(21.4%)	28(39.4%)	29(30.5%)						
	++	2(1.1%)	0	0	1(7.1%)	30(42.3%)	55(57.9%)						

	-	174(100%)	42(95.5%)	117(96.7%)	18(100%)	76(93.8%)	90(91.8%)						
SPARC in epithelial cells	+	0	2(4.5%)	4(3.3%)	0	3(3.7%)	3(3.1%)	------	------	------	------	------	------
	++	0	0	0	0	2(2.5%)	5(5.1%)						

*Numbers of valid (informative) cases.

**Including cases of only basal cells positive labeling.

***Only basal membrane positive labeling in normal epithelia.

#Low: Mild & Moderate DYS; ##High: Severe DYS & CIS.

^a^Esophageal normal epithelia *vs*. ESCC. ^b^Esophageal normal epithelia *vs*. Mild & Moderate DYS. ^c^Mild & Moderate DYS *vs*. Severe DYS & CIS. ^d^Severe DYS & CIS *vs*. ESCC stage I. ^e^ESCC stage I *vs*. ESCC stage II. ^f^ESCC stage II *vs*. ESCC stage III & IV.

**Table 3:** Summary of correlation of immunohistochemical abnormalities with clinicopathologic characteristics in ESCC

		Sex			Age			Differentiation			
		
Protein		Male	Female	*P*	60	<60	*P*	well	moderate	poor	*P*
	-	92	20		53	59		16	56	40	
Fas	+	55	10	0.053	33	32	0.088	23	29	13	0.002
	++	8	6		11	3		7	5	2	

	-	80	15		46	49		26	47	22	
FADD	+	59	20	0.278	40	39	0.781	21	33	25	0.043
	++	19	4		13	10		0	15	8	

	-	49	14		38	25		17	29	17	
CDC25B	+	89	18	0.480	47	60	0.103	25	49	33	0.852
	++	20	7		15	12		5	15	7	

	-	23	11		21	13		6	12	16	
Fascin	+	66	12	0.114	37	41	0.347	18	35	25	0.028
	++	68	16		41	43		23	46	15	

	-	59	16		38	37		6	27	42	
CK14	+	35	4	0.241	20	19	0.977	11	21	7	<0.001
	++	64	19		41	42		31	44	8	

	-	90	16		50	56		17	53	36	
CK4	+	66	20	0.242	49	37	0.018	27	39	20	0.056
	++	4	2		0	6		3	2	1	

	-	92	0		49	63		22	51	39	
Annexin 1	+	52	14	0.843	41	25	0.059	21	30	12	0.085
	++	15	4		9	10		5	12	5	

	-	39	15		26	28		11	16	27	
Laminin-5γ2	+	90	18	0.191	59	49	0.425	32	57	19	<0.001
	++	28	5		14	19		4	19	10	

	-	22	12		14	20		5	14	15	
SPARC in stromal fibroblasts	+	48	12	0.031	26	34	0.079	11	27	22	0.004
	++	74	12		51	35		28	45	13	

	-	146	38		97	87		44	88	52	
SPARC in epithelial cells	+	5	1	0.397	1	5	0.108	1	3	2	0.866
	++	7	0		2	5		2	2	3	

**Table 4:** Summary of correlation of immunohistochemical abnormalities with survival

Protein	Expression	Disease-free survival	
		
		5-year survival rate (%)	*P*
	-/+	38.16	
Fas	++	36.36	0.7803

	-/+	41.88	
FADD	++	11.11	0.0022

	-/+	40.87	
CDC25B	++	38.82	0.7252

	-/+	40.56	
Fascin	++	38.14	0.6481

	-/+	33.81	
CK14	++	44.41	0.4061

	-/+	33.54	
CK4	++	44.44	0.2903

	-/+	40.74	
Annexin 1	++	0	0.1617

	-/+	42.65	
Laminin-5γ2	++	22.22	0.0380

	-/+	48.80	
SPARC in stromal fibroblasts	++	30.97	0.0494

**Table 5:** The correlation of the expression of fascin, CK14 and laminin-5γ2 observed in Western blot with the differentiation of the primary tumor and trans-well cell migration assay of 12 ESCC cell lines

Cell lines	Differentiation	Trans-well cell migration	Fascin	CK14	Laminin-5γ2
EC9706	well	strongest	high	negative	negative
TE12	unknown	weakest	negative	negative	negative
COLO-680N	unknown	strong	high	negative	high
KYSE510	well	strong	high	negative	high
KYSE450	well	strong	high	negative	negative
KYSE410	poor	strong	high	negative	moderate
KYSE180	well	strong	high	high	moderate
KYSE150	poor	weak	high	negative	high
KYSE140	moderate	weak	high	negative	high
KYSE70	poor	weak	high	negative	moderate
KYSE30	well	weak	high	negative	negative
YES2	unknown	weak	high	negative	negative

## References

[B1] McCann J (1999). Esophageal cancers: changing character, increasing incidence. J Natl Cancer Inst.

[B2] Law SY, Fok M, Cheng SW, Wong J (1992). A comparison of outcome after resection for squamous cell carcinomas and adenocarcinomas of the esophagus and cardia. Surg Gynecol Obstet.

[B3] Nabeya K, Nakata Y (1997). Extent of resection and lymphadenectomy in early squamous cell esophageal cancer. Dis Esophagus.

[B4] Su H, Hu N, Shih J, Hu Y, Wang QH, Chuang EY, Roth MJ, Wang C, Goldstein AM, Ding T, Dawsey SM, Giffen C, Emmert-Buck MR, Taylor PR (2003). Gene expression analysis of esophageal squamous cell carcinoma reveals consistent molecular profiles related to a family history of upper gastrointestinal cancer. Cancer Res.

[B5] Zhou J, Liu Z, Wang X, Zhou C, Zhao J, Zhang R, Wu M (1999). [Gene expression profiles in squamous esophageal cancer tissues and adjacent almost normal tissues]. Zhonghua Yi Xue Yi Chuan Xue Za Zhi.

[B6] Kan T, Shimada Y, Sato F, Maeda M, Kawabe A, Kaganoi J, Itami A, Yamasaki S, Imamura M (2001). Gene expression profiling in human esophageal cancers using cDNA microarray. Biochem Biophys Res Commun.

[B7] Luo A, Kong J, Hu G, Liew CC, Xiong M, Wang X, Ji J, Wang T, Zhi H, Wu M, Liu Z (2004). Discovery of Ca2+-relevant and differentiation-associated genes downregulated in esophageal squamous cell carcinoma using cDNA microarray. Oncogene.

[B8] Zhou J, Zhao LQ, Xiong MM, Wang XQ, Yang GR, Qiu ZL, Wu M, Liu ZH (2003). Gene expression profiles at different stages of human esophageal squamous cell carcinoma. World J Gastroenterol.

[B9] Hu YC, Lam KY, Law S, Wong J, Srivastava G (2001). Identification of differentially expressed genes in esophageal squamous cell carcinoma (ESCC) by cDNA expression array: overexpression of Fra-1, Neogenin, Id-1, and CDC25B genes in ESCC. Clin Cancer Res.

[B10] Hu YC, Lam KY, Law S, Wong J, Srivastava G (2001). Profiling of differentially expressed cancer-related genes in esophageal squamous cell carcinoma (ESCC) using human cancer cDNA arrays: overexpression of oncogene MET correlates with tumor differentiation in ESCC. Clin Cancer Res.

[B11] Kononen J, Bubendorf L, Kallioniemi A, Barlund M, Schraml P, Leighton S, Torhorst J, Mihatsch MJ, Sauter G, Kallioniemi OP (1998). Tissue microarrays for high-throughput molecular profiling of tumor specimens. Nat Med.

[B12] Hao XP, Pretlow TG, Rao JS, Pretlow TP (2001). Beta-catenin expression is altered in human colonic aberrant crypt foci. Cancer Res.

[B13] Moriya Y, Niki T, Yamada T, Matsuno Y, Kondo H, Hirohashi S (2001). Increased expression of laminin-5 and its prognostic significance in lung adenocarcinomas of small size. An immunohistochemical analysis of 102 cases. Cancer.

[B14] Koukourakis MI, Giatromanolaki A, Brekken RA, Sivridis E, Gatter KC, Harris AL, Sage EH (2003). Enhanced expression of SPARC/osteonectin in the tumor-associated stroma of non-small cell lung cancer is correlated with markers of hypoxia/acidity and with poor prognosis of patients. Cancer Res.

[B15] Tong T, Zhong Y, Kong J, Dong L, Song Y, Fu M, Liu Z, Wang M, Guo L, Lu S, Wu M, Zhan Q (2004). Overexpression of Aurora-A contributes to malignant development of human esophageal squamous cell carcinoma. Clin Cancer Res.

[B16] Shimada Y, Imamura M, Wagata T, Yamaguchi N, Tobe T (1992). Characterization of 21 newly established esophageal cancer cell lines. Cancer.

[B17] Han Y, Wei F, Xu X, Cai Y, Chen B, Wang J, Xia S, Hu H, Huang X, Han Y, Wu M, Wang M (2002). [Establishment and comparative genomic hybridization analysis of human esophageal carcinomas cell line EC9706]. Zhonghua Yi Xue Yi Chuan Xue Za Zhi.

[B18] Nagata S (1997). Apoptosis by death factor. Cell.

[B19] Kase S, Osaki M, Adachi H, Kaibara N, Ito H (2002). Expression of Fas and Fas ligand in esophageal tissue mucosa and carcinomas. Int J Oncol.

[B20] Galaktionov K, Beach D (1991). Specific activation of cdc25 tyrosine phosphatases by B-type cyclins: evidence for multiple roles of mitotic cyclins. Cell.

[B21] Nishioka K, Doki Y, Shiozaki H, Yamamoto H, Tamura S, Yasuda T, Fujiwara Y, Yano M, Miyata H, Kishi K, Nakagawa H, Shamma A, Monden M (2001). Clinical significance of CDC25A and CDC25B expression in squamous cell carcinomas of the oesophagus. Br J Cancer.

[B22] Grothey A, Hashizume R, Sahin AA, McCrea PD (2000). Fascin, an actin-bundling protein associated with cell motility, is upregulated in hormone receptor negative breast cancer. Br J Cancer.

[B23] Swierczynski SL, Maitra A, Abraham SC, Iacobuzio-Donahue CA, Ashfaq R, Cameron JL, Schulick RD, Yeo CJ, Rahman A, Hinkle DA, Hruban RH, Argani P (2004). Analysis of novel tumor markers in pancreatic and biliary carcinomas using tissue microarrays. Hum Pathol.

[B24] Maitra A, Adsay NV, Argani P, Iacobuzio-Donahue C, De Marzo A, Cameron JL, Yeo CJ, Hruban RH (2003). Multicomponent analysis of the pancreatic adenocarcinoma progression model using a pancreatic intraepithelial neoplasia tissue microarray. Mod Pathol.

[B25] Hashimoto Y, Ito T, Inoue H, Okumura T, Tanaka E, Tsunoda S, Higashiyama M, Watanabe G, Imamura M, Shimada Y (2005). Prognostic significance of fascin overexpression in human esophageal squamous cell carcinoma. Clin Cancer Res.

[B26] Chu PG, Lyda MH, Weiss LM (2001). Cytokeratin 14 expression in epithelial neoplasms: a survey of 435 cases with emphasis on its value in differentiating squamous cell carcinomas from other epithelial tumours. Histopathology.

[B27] Takahashi H, Shikata N, Senzaki H, Shintaku M, Tsubura A (1995). Immunohistochemical staining patterns of keratins in normal oesophageal epithelium and carcinoma of the oesophagus. Histopathology.

[B28] Su L, Morgan PR, Lane EB (1996). Keratin 14 and 19 expression in normal, dysplastic and malignant oral epithelia. A study using in situ hybridization and immunohistochemistry. J Oral Pathol Med.

[B29] Gasparoni A, Fonzi L, Schneider GB, Wertz PW, Johnson GK, Squier CA (2004). Comparison of differentiation markers between normal and two squamous cell carcinoma cell lines in culture. Arch Oral Biol.

[B30] Morifuji M, Taniguchi S, Sakai H, Nakabeppu Y, Ohishi M (2000). Differential expression of cytokeratin after orthotopic implantation of newly established human tongue cancer cell lines of defined metastatic ability. Am J Pathol.

[B31] Grace MP, Kim KH, True LD, Fuchs E (1985). Keratin expression in normal esophageal epithelium and squamous cell carcinoma of the esophagus. Cancer Res.

[B32] Franchi A, Moroni M, Massi D, Paglierani M, Santucci M (2002). Sinonasal undifferentiated carcinoma, nasopharyngeal-type undifferentiated carcinoma, and keratinizing and nonkeratinizing squamous cell carcinoma express different cytokeratin patterns. Am J Surg Pathol.

[B33] Suo Z, Holm R, Nesland JM (1993). Squamous cell carcinomas. An immunohistochemical study of cytokeratins and involucrin in primary and metastatic tumours. Histopathology.

[B34] Alldridge LC, Harris HJ, Plevin R, Hannon R, Bryant CE (1999). The annexin protein lipocortin 1 regulates the MAPK/ERK pathway. J Biol Chem.

[B35] Ahn SH, Sawada H, Ro JY, Nicolson GL (1997). Differential expression of annexin I in human mammary ductal epithelial cells in normal and benign and malignant breast tissues. Clin Exp Metastasis.

[B36] Masaki T, Tokuda M, Ohnishi M, Watanabe S, Fujimura T, Miyamoto K, Itano T, Matsui H, Arima K, Shirai M, Maeba T, Sogawa K, Konishi R, Taniguchi K, Hatanaka Y, Hatase O, Nishioka M (1996). Enhanced expression of the protein kinase substrate annexin in human hepatocellular carcinoma. Hepatology.

[B37] Paweletz CP, Ornstein DK, Roth MJ, Bichsel VE, Gillespie JW, Calvert VS, Vocke CD, Hewitt SM, Duray PH, Herring J, Wang QH, Hu N, Linehan WM, Taylor PR, Liotta LA, Emmert-Buck MR, Petricoin EF (2000). Loss of annexin 1 correlates with early onset of tumorigenesis in esophageal and prostate carcinoma. Cancer Res.

[B38] Garcia Pedrero JM, Fernandez MP, Morgan RO, Herrero Zapatero A, Gonzalez MV, Suarez Nieto C, Rodrigo JP (2004). Annexin A1 down-regulation in head and neck cancer is associated with epithelial differentiation status. Am J Pathol.

[B39] Burgeson RE, Chiquet M, Deutzmann R, Ekblom P, Engel J, Kleinman H, Martin GR, Meneguzzi G, Paulsson M, Sanes J (1994). A new nomenclature for the laminins. Matrix Biol.

[B40] Pyke C, Romer J, Kallunki P, Lund LR, Ralfkiaer E, Dano K, Tryggvason K (1994). The gamma 2 chain of kalinin/laminin 5 is preferentially expressed in invading malignant cells in human cancers. Am J Pathol.

[B41] Koshikawa N, Moriyama K, Takamura H, Mizushima H, Nagashima Y, Yanoma S, Miyazaki K (1999). Overexpression of laminin gamma2 chain monomer in invading gastric carcinoma cells. Cancer Res.

[B42] Skyldberg B, Salo S, Eriksson E, Aspenblad U, Moberger B, Tryggvason K, Auer G (1999). Laminin-5 as a marker of invasiveness in cervical lesions. J Natl Cancer Inst.

[B43] Aoki S, Nakanishi Y, Akimoto S, Moriya Y, Yoshimura K, Kitajima M, Sakamoto M, Hirohashi S (2002). Prognostic significance of laminin-5 gamma2 chain expression in colorectal carcinoma: immunohistochemical analysis of 103 cases. Dis Colon Rectum.

[B44] Kohlberger P, Beneder C, Horvat R, Leodolter S, Breitenecker G (2003). Immunohistochemical expression of laminin-5 in cervical intraepithelial neoplasia. Gynecol Oncol.

[B45] Lenander C, Roblick UJ, Habermann JK, Ost A, Tryggvason K, Auer G (2003). Laminin 5 gamma 2 chain expression: a marker of early invasiveness in colorectal adenomas. Mol Pathol.

[B46] Porter PL, Sage EH, Lane TF, Funk SE, Gown AM (1995). Distribution of SPARC in normal and neoplastic human tissue. J Histochem Cytochem.

[B47] Le Bail B, Faouzi S, Boussarie L, Guirouilh J, Blanc JF, Carles J, Bioulac-Sage P, Balabaud C, Rosenbaum J (1999). Osteonectin/SPARC is overexpressed in human hepatocellular carcinoma. J Pathol.

