# Supplementary material for: Tissue microarray analysis reveals a tight correlation between protein expression pattern and progression of esophageal squamous cell carcinoma
Source: BMC Cancer. 2006 Dec 22;6:296. doi: 10.1186/1471-2407-6-296 (PMC1766359; doi:10.1186/1471-2407-6-296)
Supplement: Additional file 1 — The expression of mutant p53 protein in the tissue microarray. The expression of mutant p53 protein was an "early" event, occurring at mild and moderate DYS. [file 1471-2407-6-296-S1.doc]

We had examined mutant p53 protein using the TMA-based immunohistochemistry.

| Antibody | Clone | Dilution &  condition | Pretreatment | Source |
| --- | --- | --- | --- | --- |
| p53 | DO-7 | 1:50 RT1h | MWO-CB | Dakocytomation, Glostrup, Denmark |

RT, room temperature

MWO-CB, microwave oven heating in citrate buffer.

The criteria of scoring: negative(－), <5% cells positive; weakly positive (+), 5～50% cells positive; strongly positive (++), >50% cells positive.

**Results**


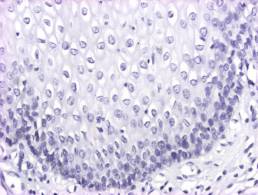

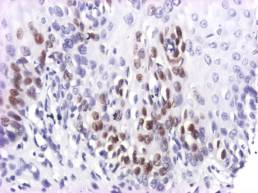

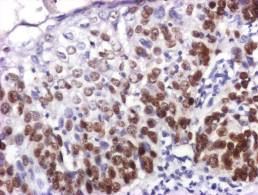

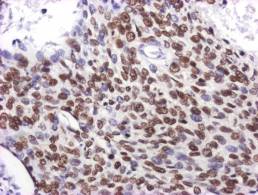


**a**

**b**

**c**

**d**

Representative photographs of immunohistochemical features of mutant p53 protein in normal esophageal mucosa (a), mild DYS (b), severe DYS (c) and ESCC (d).

Absence of mutant p53 labeling is seen in normal esophageal epithelia, but presence of nuclear mutant p53 labeling is seen in mild, severe DYS and ESCC.

| Protein | | Normal * | Precursor lesions* | | ESCC* | | | *P-value* | | | | | |
| --- | --- | --- | --- | --- | --- | --- | --- | --- | --- | --- | --- | --- | --- |
|  |  |  | Low# | High## | I | IIA/IIB | III/IV | *P*^a^ | *P*^b^ | *P*^c^ | *P*^d^ | *P*^e^ | *P*^f^ |
| p53 | － | 131(78.4%) | 15(38.5%) | 52(43.7%) | 5(29.4%) | 51(63.8%) | 53(53.5%) | <0.001 | <0.001 | 0.832 | 0.050 | 0.004 | 0.065 |
|  | + | 30(18.0%) | 12(30.8%) | 32(26.9%) | 2(11.8%) | 13(16.3%) | 31(31.3%) |  |  |  |  |  |  |
|  | ++ | 6(3.6%) | 12(30.8%) | 35(29.4%) | 10(58.9%) | 16(20%) | 15(15.2%) |  |  |  |  |  |  |

*Numbers of valid (informative) cases.

#Low: Mild & Moderate DYS; ##High: Severe DYS & CIS

^a^Esophageal normal epithelia *vs.* ESCC. ^b^Esophageal normal epithelia *vs.* Mild & Moderate DYS. ^c^Mild & Moderate DYS *vs.* Severe DYS & CIS. ^d^Severe DYS & CIS *vs.* ESCC stage I. ^e^ESCC stage I *vs.* ESCC stage II. ^f^ESCC stage II *vs.* ESCC stage III &IV.

The results showed that expression of mutant p53 protein was an "early" event, occurring at mild and moderate DYS.

**Discussion**

**Mutant protein of Tumor Suppressor Gene (p53)**

P53 is a tumor suppressor gene that has important roles in growth arrest, apoptosis, DNA repair and cell differentiation. It is also well known that mutation of p53 gene is one of the most common genetic alterations in human cancer. The mutated of p53 gene will produce mutant p53 protein which loses the function of wild type and results in cell growth out of control [1]. Immunohistochemical detection of nuclear p53 protein can act as a surrogate marker of mutation of p53 gene.

In the present study as well as previous studies, mutant p53 protein was detected not only in ESCC but also in esophageal precursor lesions [2]. In addition, our results confirmed that p53 mutation, as assessed by nuclear overexpression of p53 protein, was an “early” event in the progression of ESCC, occurring at the mild and moderate DYS lesions.

REFERENCES

1. Nishioka H, Hiasa Y, Hayashi I, Kitahori Y, Konishi N, Sugimura M: **Immunohistochemical detection of p53 oncoprotein in human oral squamous cell carcinomas and leukoplakias: comparison with proliferating cell nuclear antigen staining and correlation with clinicopathological findings**. *Oncology* 1993, **50**(6):426-429.

2. Yasuda M, Kuwano H, Watanabe M, Toh Y, Ohno S, Sugimachi K: **p53 expression in squamous dysplasia associated with carcinoma of the oesophagus: evidence for field carcinogenesis**. *Br J Cancer* 2000, **83**(8):1033-1038.
